# Comparative Genomics Reveals Two Novel RNAi Factors in *Trypanosoma brucei* and Provides Insight into the Core Machinery

**DOI:** 10.1371/journal.ppat.1002678

**Published:** 2012-05-24

**Authors:** Rebecca L. Barnes, Huafang Shi, Nikolay G. Kolev, Christian Tschudi, Elisabetta Ullu

**Affiliations:** 1 Department of Internal Medicine, Yale University, New Haven, Connecticut, United States of America; 2 Division of Epidemiology of Microbial Diseases, School of Public Health, Yale University, New Haven, Connecticut, United States of America; 3 Department of Cell Biology, School of Medicine, Yale University, New Haven, Connecticut, United States of America; University of Wisconsin-Madison, United States of America

## Abstract

The introduction ten years ago of RNA interference (RNAi) as a tool for molecular exploration in *Trypanosoma brucei* has led to a surge in our understanding of the pathogenesis and biology of this human parasite. In particular, a genome-wide RNAi screen has recently been combined with next-generation Illumina sequencing to expose catalogues of genes associated with loss of fitness in distinct developmental stages. At present, this technology is restricted to RNAi-positive protozoan parasites, which excludes *T. cruzi*, *Leishmania major*, and *Plasmodium falciparum*. Therefore, elucidating the mechanism of RNAi and identifying the essential components of the pathway is fundamental for improving RNAi efficiency in *T. brucei* and for transferring the RNAi tool to RNAi-deficient pathogens. Here we used comparative genomics of RNAi-positive and -negative trypanosomatid protozoans to identify the repertoire of factors in *T. brucei*. In addition to the previously characterized Argonaute 1 (AGO1) protein and the cytoplasmic and nuclear Dicers, *Tb*DCL1 and *Tb*DCL2, respectively, we identified the RNA Interference Factors 4 and 5 (*Tb*RIF4 and *Tb*RIF5). *Tb*RIF4 is a 3′-5′ exonuclease of the DnaQ superfamily and plays a critical role in the conversion of duplex siRNAs to the single-stranded form, thus generating a *Tb*AGO1-siRNA complex required for target-specific cleavage. *Tb*RIF5 is essential for cytoplasmic RNAi and appears to act as a *Tb*DCL1 cofactor. The availability of the core RNAi machinery in *T. brucei* provides a platform to gain mechanistic insights in this ancient eukaryote and to identify the minimal set of components required to reconstitute RNAi in RNAi-deficient parasites.

## Author Summary

RNA interference (RNAi), a naturally-occurring pathway whereby the presence of double-stranded RNA in a cell triggers the degradation of homologous mRNA, has been harnessed in many organisms as an invaluable molecular biology tool to interrogate gene function. Although this technology is widely used in the protozoan parasite *Trypanosoma brucei*, other parasites of considerable public health significance, such as *Trypanosoma cruzi*, *Leishmania major*, and *Plasmodium falciparum* do not perform RNAi. Since RNAi has recently been introduced into budding yeast, this opens up the possibility that RNAi can be reconstituted in these pathogens. The key to this is getting a handle on the essential RNAi factors in *T. brucei*. By applying comparative genomics we identified five genes that are present in the RNAi-proficient species, but not in RNAi-deficient species: three previously identified RNAi factors, and two novel ones, which are described here. This insight into the core *T. brucei* RNAi machinery represents a major step towards transferring this pathway to RNAi-deficient parasites.

## Introduction

RNA interference (RNAi) was first described in 1998 and within a short period of time tremendously facilitated the analysis of gene function, especially in organisms where classical genetic approaches are not available. This is particularly evident in the human pathogen *Trypanosoma brucei*, where RNAi has become a primary tool to interrogate its biology aided by the availability of an inducible and heritable system [1], [2]. The effectiveness of RNAi in *T. brucei* is documented by over 500 publications in the past ten years and the availability of the genome sequence has opened up the possibility for genome-wide RNAi screens [1]. In particular, the very recently introduced RIT-Seq method (RNA Interference Target Sequencing) took advantage of the power of genome-wide RNAi screens and combined it with the strength and depth of next-generation Illumina sequencing [3]. This strategy provided the scientific community with a catalogue of genes whose knock-down is detrimental to the parasite under a variety of developmental conditions and is likely to find numerous applications in RNAi-positive parasites.

Although RNAi has flourished immensely in *T. brucei*, as highlighted by the above brief synopsis, it was rather disappointing to recognize both experimentally and at the genome sequence level that other protozoan parasites with a major impact on mankind, including *T. cruzi* [4], *Leishmania major*, *L. donovani* [5] and *Plasmodium falciparum* [6], were lacking a functional RNAi pathway. On the other hand, the genome sequence of *L. (Viannia) braziliensis* [7] predicted the existence of the RNAi pathway in this leishmania subgenus, and this prediction was recently experimentally validated [8]. The realization that *T. cruzi* and old world leishmanias are RNAi-negative was quite unfortunate, but it has been argued that once the core genes involved in *T. brucei* RNAi are identified it might be possible to try to reconstruct the pathway in RNAi-negative trypanosomatids [1]. Indeed, the recent success of David Bartel's group in reconstructing the RNAi pathway in *S. cerevisiae* [9] provides a proof of principle and informs us that it is realistic to try to achieve the same goal in *T. cruzi* or old world *Leishmania sp*.

Comparison of RNAi mechanisms in different model organisms [10] suggests that the common, minimal machinery consisted of firstly, a Dicer endonuclease of the RNase III family that processes long dsRNAs into duplex small interfering RNAs (siRNAs) with characteristic two-nucleotide 3′ overhangs; secondly, an Argonaute (AGO) “slicer” endonuclease that in a complex with a single-stranded (ss) guide siRNA (as opposed to the passenger strand that is discarded) cleaves target mRNA; and thirdly, a Dicer cofactor with a dsRNA-binding domain that facilitates siRNA biogenesis and loading into AGO. The AGO-siRNA complex forms the catalytic engine of the RNA-induced silencing complex or RISC [11]. An additional factor found only in some RNAi-proficient organisms is an RNA-dependent RNA polymerase [12] that creates secondary siRNAs to amplify the initial RNAi response.

The players identified so far in *T. brucei* RNAi are a single AGO slicer, *Tb*AGO1 [13], [14], and two Dicer homologs, *Tb*DCL1 [15] and *Tb*DCL2 [16]. This set of genes is also present in *L. (V) braziliensis* [8], although their precise function needs to be experimentally addressed. In *T. brucei* we have provided evidence that the nuclear Dicer *Tb*DCL2 is the first line of defence against dsRNAs originating from retroposons (ingi and SLACS) and satellite-like repeats (CIR147), whereas in the cytoplasm *Tb*DCL1 processes intermediate-sized dsRNA molecules generated by *Tb*DCL2, as well as dsRNA that may escape from the nucleus or enter the cytoplasm from the exterior milieu [15], [16]. The RISC loading mechanism in *T. brucei* is not known; no RNAi-specific dsRNA-binding protein has been identified yet [17]. In Drosophila [18], [19] and Neurospora [20] it has been shown that mutations of AGO slicer catalytic residues prevent cleavage of both the siRNA passenger strand and the target RNA, and siRNAs associated with the corresponding AGO are double-stranded. In contrast, mutations that affect target RNA degradation by *Tb*AGO1 *in vivo* do not affect the maturation of siRNAs from duplex to single-stranded form and *Tb*AGO1 is associated with single-stranded siRNAs [21], [22].

The above observations hinted at an additional activity involved in the formation of the *Tb*AGO1/ss-siRNA complex. To test this hypothesis we compared the genomes of RNAi-proficient trypanosomatid protozoa with those that have lost the RNAi machinery. In addition to the three characterized RNAi proteins in *T. brucei*, we identified two novel factors, both of which we have shown to be active in RNAi. RNA Interference Factor 5 (*Tb*RIF5) is required for cytoplasmic, but not nuclear RNAi, and appears to act in conjunction with *Tb*DCL1. *Tb*RIF4 contains a C-terminal domain related to 3′-5′ exonucleases of the DnaQ superfamily. In the absence of *Tb*RIF4 protein or function, duplex siRNAs accumulate, and are not associated with *Tb*AGO1. Human AGO2 can replace both *Tb*RIF4 and *Tb*AGO1 functions, suggesting that the slicing function of human AGO2 in RISC maturation is replaced in trypanosomes by the *Tb*RIF4 exonuclease.

## Results

### *Tb*RIF4 and *Tb*RIF5 are two novel *T. brucei* RNAi factors identified by comparative genomics

In order to identify the core RNAi genes we compared the genomes of RNAi-deficient (*T. cruzi* and *L. major*) and RNAi-proficient (*T. brucei*, *T. congolense* and *L. braziliensis*) trypanosomatids and selected ORFs that are exclusively present in all the RNAi-positive organisms, but absent from the RNAi-negative parasites. This analysis only returned five genes. In addition to *Tb*AGO1, *Tb*DCL1 and *Tb*DCL2, the search identified two putative RNAi factors (Tb927.10.8880 and Tb927.10.10730 in *T. brucei*), which we named *T. brucei* RNA Interference Factor 4 and 5 (*Tb*RIF4, 490 amino acids long, and *Tb*RIF5, 1509 amino acids long).

**Figure 1:**
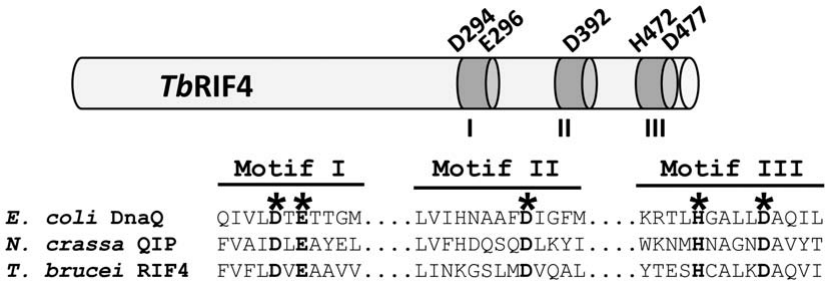
Characteristics of *Tb*RIF4. Schematic representation of *Tb*RIF4 (the drawing is not to scale) and alignment of the three motifs characteristic of 3′-5′ exonucleases of the DEDDh superfamily from *E. coli* DnaQ [25], *N. crassa* QIP [20], and *T. brucei* RIF4. Asterisks and bold characters indicate conserved residues.

At the primary sequence level the C-terminus of *Tb*RIF4 contains a domain that resembles the DnaQ superfamily of 3′-5′ exonucleases (Figure 1). This structural feature is shared with *Neurospora crassa* QIP (QDE2 Interacting Protein), which functions in removal of the passenger strand siRNA fragments, after it has been cleaved by the AGO slicer *Nc*QDE2 [20]. This protein family is characterized by three motifs encompassing the active site residues DEDDh that are present in *Tb*RIF4, as noted in a bioinformatic screen by Trudeau and colleagues [23]. The active site residues are conserved in all RIF4 homologs identified in RNAi-proficient trypanosomatids (Figure S1A) and using the alignment interface of SWISS-MODEL [24], structural predictions (Figure S1B) revealed significant similarity with the exonuclease domain of DnaQ [25]. Besides the exonuclease motifs, *Tb*RIF4 and *Nc*QIP do not share any other sequence similarity. *Tb*RIF5 is specific to the trypanosomatid lineage and bioinformatic analysis did not reveal any known domains (Figure S2).

**Figure 2:**
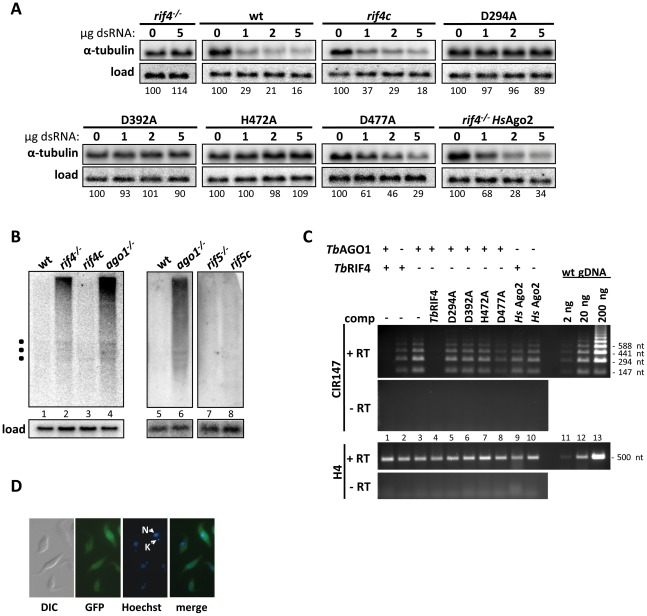
Analysis of *Tb*RIF4- and *Tb*RIF5-mutant cell lines. (*A*) Response of *rif4^−/−^* cells and cells expressing *Tb*RIF4 catalytic mutants or *Hs*AGO2 to transfection of α-tubulin dsRNA. Cells were electroporated with different amounts (in micrograms) of α-tubulin dsRNA, as indicated above each lane or with 5 µg poly(I-C) (first lane in each panel set), and total RNA was prepared 2 h after electroporation and analyzed by Northern hybridization with an α-tubulin-specific probe (α-tubulin panels). α-tubulin mRNA hybridization was quantitated by PhosphorImager analysis, normalized to the load control and expressed as % mRNA remaining, setting as 100% the amount of α-tubulin mRNA present in the samples that received poly(I-C). Load; hybridization to fumarate hydratase mRNA. (*B*) Steady-state levels of repeat-derived (CIR147) transcripts are increased in *rif4^−/−^* cells but not *rif5^−/−^* cells. Total RNA isolated from various cell lines, as indicated above each lane, was analyzed by Northern hybridization with a CIR147-specific probe. Load; hybridization to α-tubulin mRNA. Filled squares indicate the positions of the large ribosomal RNAs, as determined by methylene blue staining of the membranes. (*C*) Semi-quantitative RT-PCR analysis of CIR147 transcript levels. cDNA derived from various cell lines (as indicated above each lane; lanes 1–10) was used as a template for 22 cycles of PCR using oligonucleotides that amplify the CIR147 tandem repeat transcripts, producing a ladder of fragments (top two panels), or a portion of histone H4 (bottom two panels). Dilutions of genomic DNA were used as a positive control and an indicator for non-saturated PCR (lanes 11–13); mock cDNA synthesis without reverse transcriptase (second and fourth panels, -RT) served as a negative control. *(D)* Cells were visualized using differential interference microscopy (DIC), GFP imaging, and Hoechst (Nucleus and Kinetoplast are indicated by N and K, respectively); GFP and Hoechst images were merged (merge).

*Tb*RIF4 is essential for RNAi in both the cytoplasm and the nucleus. First, *rif4^−/−^* cells (Figure S3A) had a much reduced ability to respond to transfection with α-tubulin dsRNA (Table 1, Figure 2A), an assay that monitors the cytoplasmic RNAi response and in wild-type cells results in degradation of α-tubulin mRNA. Second, in *rif4^−/−^* cells the levels of long and heterogeneous repeat-derived (CIR147) transcripts were substantially increased as compared to wild-type cells (Figure 2B, lane 2, and Figure 2C, lane 3), a previously-reported hallmark of nuclear RNAi deficiency [16]. A *Tb*RIF4-GFP cassette, integrated into the tubulin locus of *rif4^−/−^* cells (*rif4c* cell line, Figure S3A), restored *Tb*RIF4 levels and complemented the mutant phenotypes, leading to wild-type response to dsRNA transfection (Table 1, Figure 2A) and restoration of long retroposon- and repeat-derived transcript levels (Figure 2B, lane 3, and Figure 2C, lane 4). Consistent with the involvement of *Tb*RIF4 in both the nuclear and cytoplasmic arms of the RNAi pathway, the *Tb*RIF4-GFP fusion protein was distributed uniformly between the nucleus and cytoplasm (Figure 2D).

**Table 1:** Analysis of cytoplasmic RNAi in *rif4^−/−^* and *rif5^−/−^* cell lines.

	dsRNA			
Cell line	0 µg	1 µg	2 µg	5 µg
**wt**	<5	39	56	100
***rif4^−/−^***	<5	<5	<5	<5
***rif4c***	<5	40	47	94
***rif5^−/−^***	<5	<5	<5	<5
***rif5c***	<5	25	37	93
***ago1^−/−^***	<5	<5	<5	<5

Cell lines were transfected with the indicated amounts of dsRNA. The FAT cell phenotype was scored 16 hr post-transfection and is represented as % FAT cells; the response of wild-type cells transfected with 5 µg dsRNA was set at 100% and the values for the other cell lines were normalized accordingly. For each cell line the experiments were repeated three times, and the average value is shown.

*Tb*RIF5, on the other hand, only appears to be required for the cytoplasmic branch of the RNAi pathway. *rif5^−/−^* cells (Figure S3B) transfected with double-stranded α-tubulin RNA showed a severe defect in the cytoplasmic RNAi response (Table 1). However, levels of CIR147 transcripts were not increased by *Tb*RIF5 ablation (Figure 2B, lane 7), suggesting that nuclear RNAi was unaffected. A *Tb*RIF5-BB2-HA cassette, integrated into the tubulin locus of *rif5^−/−^* cells (*rif5c* cell line, Figure S3B), complemented the cytoplasmic RNAi defect (Table 1). Due to the low level of expression of the epitope-tagged protein, the cellular localization of *Tb*RIF5 could not be ascertained by IFA. However, cell fractionation experiments indicated that most of the protein partitioned in the cytoplasm (data not shown).

### *Tb*RIF4 functions downstream of the dicer step of RNAi, while *Tb*RIF5 acts with *Tb*DCL1

**Figure 3:**
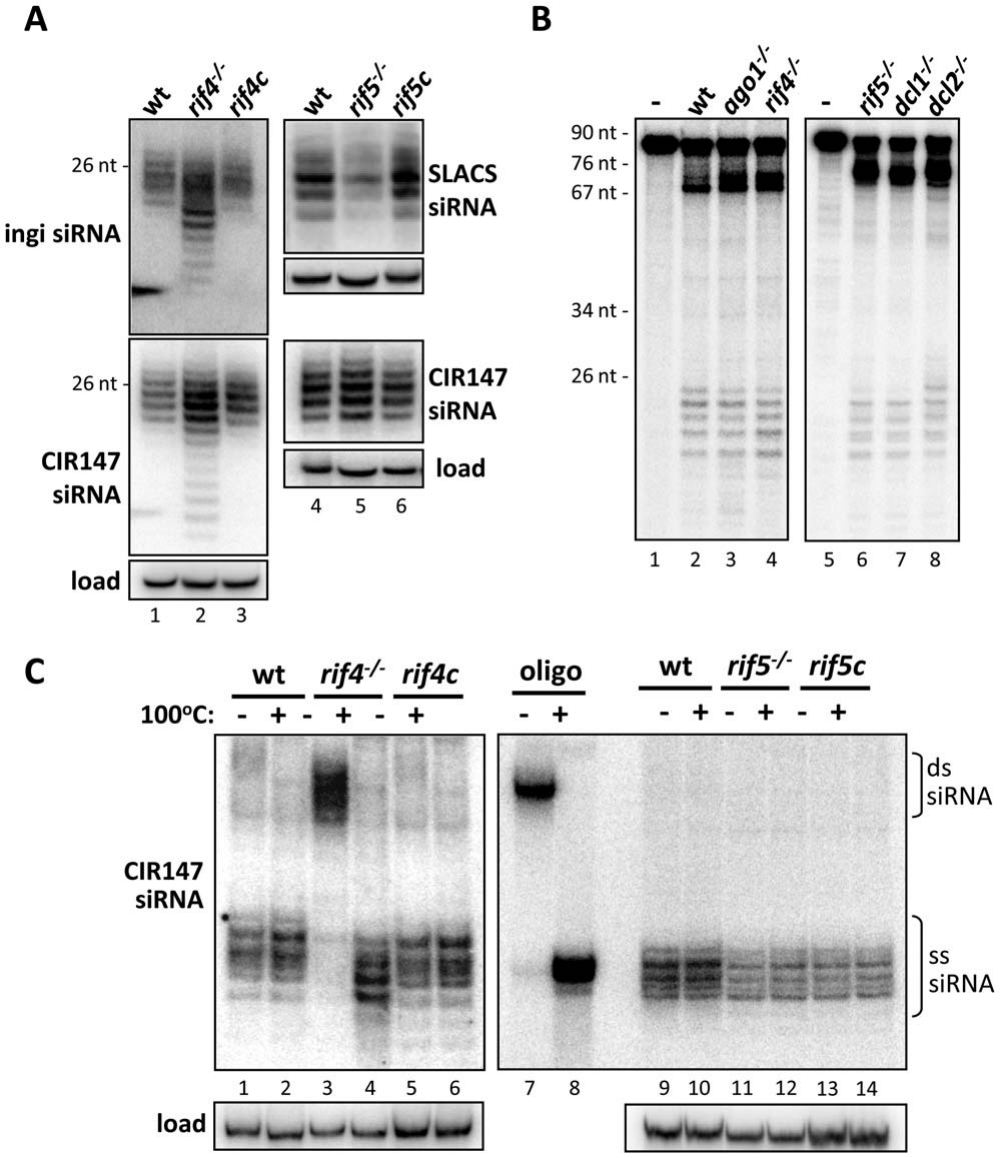
Analysis of siRNAs in *rif4^−/−^* and *rif5^−/−^* backgrounds. *(A)* Steady-state siRNA levels in *rif4^−/−^* and *rif5^−/−^* cells. Low-molecular weight RNAs were separated by denaturing gel electrophoresis and analyzed by Northern hybridization with an ingi-, SLACS- or CIR147-specific probe, as indicated next to each panel. Load; hybridization to 5S rRNA. 26 nt, DNA marker. (*B*) Dicing is unaffected in *ago1^−/−^* and *rif4^−/−^ T. brucei* extracts, but shows an altered pattern in *rif5^−/−^* extracts. Whole cell extracts from cell lines, as indicated above the lanes, or buffer alone (−) were incubated with a 83-nt, internally labelled dsRNA substrate. After incubation the products of digestion were separated on a denaturing polyacrylamide gel. Size marker positions are indicated. (*C*) Native gel analysis of siRNAs from *rif4^−/−^* and *rif5^−/−^* cells. Low molecular weight RNAs isolated from various cell lines, as indicated above each lane, were resolved on a native polyacrylamide gel without (−) or with (+) heating the samples to 100°C for 2 minutes prior to electrophoresis and analyzed by Northern blotting with a CIR147 probe. A radiolabelled synthetic RNA was included as a control (lanes 7 and 8). Load; hybridization to 5S rRNA.

To determine the stage in the RNAi pathway at which *Tb*RIF4 and *Tb*RIF5 act, we first asked whether siRNAs were produced in their absence. Northern blot analysis revealed that in *rif4^−/−^* cells (Figure 3A, lane 2) ingi- and CIR147-derived siRNAs accumulated to levels greater than wild-type (lane 1) or *rif4c* (lane 3) trypanosomes, although their size distribution was more heterogeneous than in wild-type cells, possibly due to nuclease nibbling as these siRNAs are not associated with *Tb*AGO1 (see below). An *in vitro* dicing assay programmed with long dsRNA [16] indicated that siRNA processing was as active in *rif4^−/−^* as in wild-type cell extracts (Figure 3B), suggesting that *Tb*RIF4 acts downstream of the dicing step.

siRNAs also accumulated in *rif5^−/−^* parasites. However, as in a *dcl1^−/−^* cell line [16], while siRNAs derived from the CIR147 tandem repeats accumulated in *rif5^−/−^* cells to a level comparable to wild-type or *rif5c* trypanosomes (Figure 3A), SLACS retroposon siRNA levels were reduced in the mutant cell line (lane 5), but were restored by complementation (lane 6). A similar phenotype was observed for the ingi-derived siRNAs (data not shown). These data suggested a link to the cytoplasmic Dicer and the following experiments provided corroborating evidence. Firstly, the *in vitro* dicing assay showed that the siRNAs generated by a *rif5^−/−^* cell extract were the same size as those created by a *dcl1^−/−^* extract, which, as has been shown previously by our group, are one nucleotide smaller than those generated by a *dcl2^−/−^* extract ([16]; Figure 3B). Secondly, we co-immunoprecipitated the two proteins (Figure S4) and found that a proportion of *Tb*DCL1 is in a complex with *Tb*RIF5. We expressed *Tb*DCL1-BB2-FLAG and *Tb*RIF5-BB2-HA in the same cell line. Immunoprecipitation of *Tb*DCL1 (top panel) or *Tb*RIF5 (bottom panel) was carried out using anti-FLAG or anti-HA antibodies, respectively, and the fate of both *Tb*DCL1 and *Tb*RIF5 was monitored by Western blotting for the common BB2 epitope. It should be noted that *Tb*DCL1-BB2-FLAG is at least two to three times more abundant than *Tb*RIF5-BB2-HA in this cell line (see input lane in Figure S4). When *Tb*DCL1 was pulled down, the majority of *Tb*RIF5 was found in the immunoprecipitated fraction. In contrast, when *Tb*RIF5 was immunoprecipitated, approximately 50% of *Tb*DCL1 was detected in the precipitate, in agreement with the lower abundance of *Tb*RIF5 (the identity of the immunoprecipitated proteins was verified by Western blotting with HA monoclonal antibody for *Tb*RIF5, or FLAG monoclonal antibody for *Tb*DCL1; data not shown). The interaction of these two factors was further supported by: i. co-fractionation on a Superdex-200 sizing column; ii. mass-spectrometry of FLAG-immunoprecipitated *Tb*DCL1, which revealed several peptides derived from *Tb*RIF5 as well as *Tb*DCL1 (data not shown). Thus, we concluded that *Tb*DCL1 and *Tb*RIF5 interact either directly or indirectly.

### siRNAs in *Tb*RIF4 null trypanosomes accumulate in duplex form

An essential step in the RNAi pathway is the formation of the RNA-induced silencing complex or RISC [26], consisting of an AGO “slicer”, an endonuclease of the RNase H family, in a complex with an siRNA, which guides target cleavage by AGO. Biochemical studies have shown that human and *Drosophila* AGO2 are initially loaded with duplex siRNAs and, following passenger strand nicking by AGO, the resulting fragments dissociate [27]. Specific factors required for this transition have been identified, such as the multimeric endonuclease C3PO (Component 3 Promoter of RISC) in *Drosophila* and humans [28], [29] or the *Neurospora* 3′-5′ exonuclease QIP [20], [30], [31]. Thus, to test whether *Tb*RIF4 and/or *Tb*RIF5 were involved in RISC formation, we next asked whether the *rif4^−/−^*- and *rif5^−/−^*-derived siRNAs were single- or double-stranded. We purified RNA from wild-type, *rif4^−/−^*, *rif4c*, *rif5^−/−^*, and *rif5c* S100 extracts and analyzed the samples on a native polyacrylamide gel with or without denaturation at 100°C (Figure 3C). A radiolabelled double-stranded synthetic siRNA was used as a control (lanes 7 and 8). Following Northern blot hybridization with a CIR147 probe, most siRNAs from wild-type cells appeared single-stranded (lane 1, see [22]). A similar result was obtained with siRNAs from *rif5^−/−^* and *rif5c* cells (lanes 11 and 13). In contrast, by this analysis non-denatured *rif4^−/−^*-derived siRNAs displayed the mobility expected for duplex molecules (compare lanes 3 and 7), but were converted to ss-siRNAs upon denaturation (lane 4), whereas siRNAs from the *rif4c* sample again reproduced the single-stranded nature present in wild-type cells (lane 5).

To provide additional evidence that siRNAs isolated from *rif4^−/−^* trypanosomes were double-stranded, we incubated the RNA samples at different temperatures (20° to 100°C) prior to loading them on a native gel (Figure S5A). Ingi siRNA strands dissociated between 60°C and 65°C, consistent with a duplex structure. Lastly, we assessed the sensitivity of siRNAs isolated from wild-type and *rif4^−/−^* cells to treatment with RNase T1 and RNase A, which preferentially cleave ss RNA in high salt (Figure S5B). Wild-type CIR147 siRNAs were mostly degraded by the ribonucleases (lane 2), with trace amounts resistant to digestion, but siRNAs derived from *rif4^−/−^* cells were largely resistant to degradation (lane 5) and were only cleaved efficiently following denaturation (lane 6).

The above results strongly indicated that *rif4^−/−^* siRNAs are predominantly in duplex form. We think it is unlikely that the duplex siRNAs are an artefact of the RNA isolation procedure, since very little duplex siRNA can be observed in wild-type samples prepared under the same conditions (Figure 3C, lane 1). We showed previously by Northern blotting [32] that both sense and antisense siRNAs from the retroposons ingi and SLACS accumulate in *T. brucei*. Recent deep-sequencing of siRNAs associated with *Tb*AGO1 confirmed these early observations and extended them to include CIR147 (unpublished observations). Although wild-type siRNAs have the potential to anneal to form duplex structures, they do not appear to do so under the experimental conditions used.

### siRNAs are not associated with *Tb*AGO1 in *Tb*RIF4 null cells

**Figure 4:**
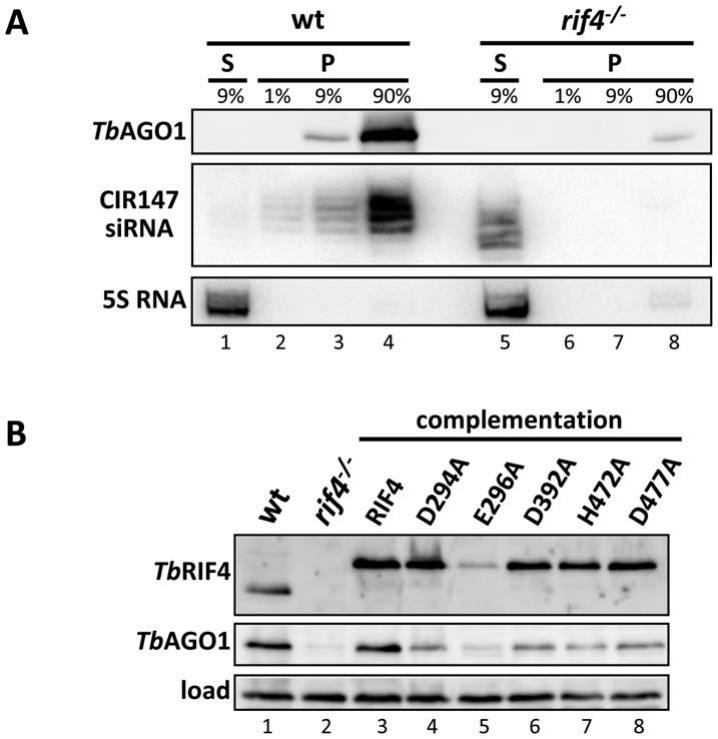
siRNAs are not associated with *Tb*AGO1 in *rif4^−/−^* cells. (*A*) Cytoplasmic extracts from cells expressing TAP-tagged *Tb*AGO1 in a wild-type (lanes 1–4) or *rif4^−/−^* (lanes 5–8) background were immunoprecipitated with IgG beads, and the indicated amounts of supernatant (S, lanes 1 and 5) and immunoprecipitated material (P, lanes 2–4 and 6–8) were analyzed by Western blotting with a polyclonal anti-*Tb*AGO1 antibody (upper panel, *Tb*AGO1). CIR147 siRNAs in the soluble and precipitated materials were revealed by Northern hybridization (middle panel, CIR147 siRNA). RNA loading was quantified by hybridization to 5S rRNA (bottom panel, 5S RNA). (*B*) *Tb*RIF4 and *Tb*AGO1 steady-state levels in various cell lines. Total protein extracts from 5×10^6^ cells from various cell lines, as indicated above each lane, were resolved by SDS-PAGE and Western blotted with a polyclonal anti-*Tb*RIF4 antibody (top panel, *Tb*RIF4) or a polyclonal anti-*Tb*AGO1 antibody (middle panel, *Tb*AGO1). Load; a cross-reacting band to the *Tb*AGO1 antibody (bottom panel).

Next we asked whether siRNAs in *rif4^−/−^* cells were associated with *Tb*AGO1. To this end, we introduced TAP-tagged *Tb*AGO1, which fully complements *Tb*AGO1 deficiency [14], into *rif4^−/−^* cells. It should be noted that the level of TAP-tagged *Tb*AGO1 was much lower in *rif4^−/−^* cells as compared to wild-type trypanosomes (Figure 4A, top panel, compare lanes 4 and 8). This dramatic diminution of *Tb*AGO1 levels was also observed for endogenous *Tb*AGO1 in *rif4^−/−^* cells (Figure 4B, middle panel, compare lanes 1 and 2), but returned to normal upon complementation (lane 3). By semi-quantitative Western blot analysis we estimated that the level of endogenous *Tb*AGO1 was reduced approximately 20-fold in *rif4^−/−^* cells (data not shown).

TAP-tagged *Tb*AGO1 was immunoprecipitated from wild-type and *rif4^−/−^* S100 extracts and varying amounts from the different samples were analysed by Western (Figure 4A, top panel) or Northern blotting (middle panel). This experiment showed that when roughly similar amounts of *Tb*AGO1 protein were loaded (compare 90% of the immunoprecipitated material from *rif4^−/−^* cells with 9% or even 1% of the material from the wild-type sample), no siRNAs could be detected in the pellet from the *rif4^−/−^* sample (lanes 6–8), although these molecules were clearly visible in the wild-type immunoprecipitate (lanes 2–4). We concluded that the majority of CIR147 siRNAs in *rif4^−/−^* cells were not associated with *Tb*AGO1.

### Biochemical investigation of *Tb*RIF4 exonuclease activity

**Figure 5:**
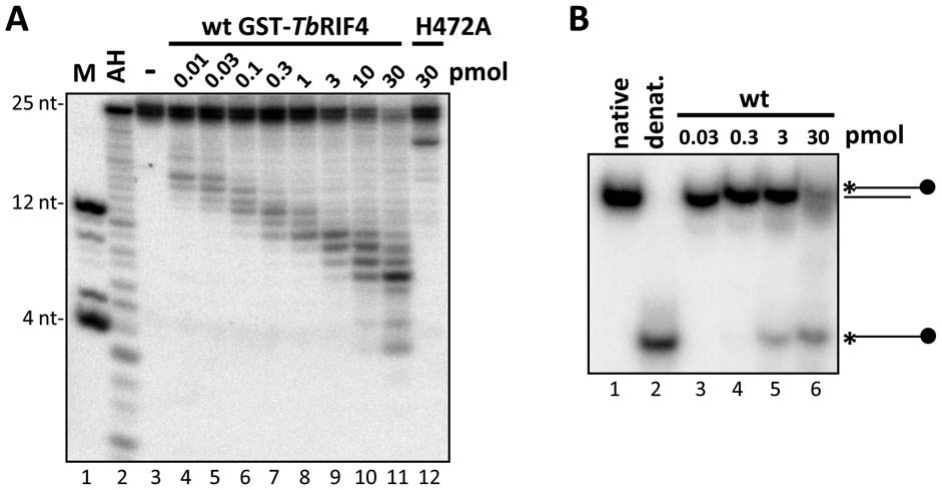
Recombinant *Tb*RIF4 3′-5′ exonuclease activity *in vitro*. (*A*) Titration of GST-RIF4 activity. 1 pmol of a 25 nt-long, 5′-end labelled synthetic dsRNA was incubated with 0.01–30 pmol GST-RIF4 (lanes 4–11; molar ratios from 10∶1 to 1∶30) or 30 pmol GST-*Tb*RIF4 carrying mutation H472A (lane 12). Lane 3, input. AH, alkaline hydrolysate ladder. M, marker oligonucleotides. Samples were separated on a denaturing 20% polyacrylamide gel. (*B*) Action of GST-*Tb*RIF4 on a RNA substrate blocked at the 3′ end of one strand. 1 pmol of a 25 nt-long, 5′-end labelled synthetic dsRNA with a dideoxycytosine residue at the 3′ end of the labelled strand was incubated with 0.03–30 pmol GST-*Tb*RIF4 (lanes 3–6), or buffer alone (lanes 1 and 2). Samples were separated on a non-denaturing 16% polyacrylamide gel. As a control for the separation of double-stranded and single-stranded species, the substrate incubated with buffer alone was resolved on the same gel without (lane 1, native) or with (lane 2, denat.) heating to 100°C for 2 minutes prior to electrophoresis.

Our experiments so far suggested that *Tb*RIF4 plays a role in the conversion of double- to single-stranded siRNAs. To test whether the predicted 3′-5′ exonuclease domain in the C-terminus of *Tb*RIF4 was involved in this process, we generated recombinant GST-*Tb*RIF4 fusion proteins (Figure S6A) and tested its activity *in vitro*. Incubation of 25-nt duplex α-tubulin siRNA-315 [16], labelled at the 5′ end of the sense strand, with increasing amounts of GST-*Tb*RIF4 produced a distinct ladder of fragments that progressively decreased in size with increasing protein concentration (Figure 5A, lanes 4–11) and with time (Figure S6B, lanes 2–7). Mutation of conserved exonuclease active site residue H472 to alanine drastically reduced the detected exonuclease activity (Figure 5A, lane 12 and Figure S6B, lane 8). With the above substrate we observed accumulation of a 7-nt fragment, but this was not the case when the antisense strand of the duplex substrate was labelled (Figure S6C), indicating that digestion by *Tb*RIF4 is somewhat affected by the substrate sequence. Nevertheless, these results suggested that *in vitro* both strands are accessible to *Tb*RIF4 digestion and that the enzyme has a distributive rather than processive mode of action.

To further characterize *Tb*RIF4 activity, we first used substrates with no, single or double 3′ nucleotide overhang (the label was placed at the 5′ end of the sense strand) and found that they were also processed, suggesting that the 2-nt 3′ extension is not important for substrate recognition by recombinant *Tb*RIF4 *in vitro* (Figure S6D), consistent with a distributive mode of action. Secondly, we showed that recombinant *Tb*RIF4 acted preferentially on duplex as opposed to single-stranded RNA (Figure S6E) and that catalysis required magnesium ions (Figure S6F) and a free 3′ end (Figure S6G), as processing of the RNA substrate was inhibited by EDTA or by blocking the 3′ end with a 2′,3′-dideoxycytosine, respectively. Thirdly, we asked whether GST-*Tb*RIF4 can convert the RNA duplex into ss-RNA. Recombinant *Tb*RIF4 does not exhibit clear strand selectivity (Figure S6C), thus either strand can be degraded and any released ssRNA will base-pair with available complementary products to form dsRNA. To circumvent this problem, we carried out an *in vitro* assay where one strand of the double-stranded RNA substrate was labelled at the 5′ end and the 3′ end of the same strand was blocked by dideoxy-C to prevent it from being a substrate for *Tb*RIF4 (Figure 5B). This mimics the effect of a bound protein protecting one end of the duplex, a likely *in vivo* scenario. The products of this reaction were separated on a native gel, showing that many of the molecules had been converted to the single-stranded form. Thus, *Tb*RIF4 has an intrinsic 3′-5′ exonuclease activity *in vitro*, which acts preferentially on double-stranded RNA, and its activity is dependent on a predicted active site residue.

### Active site residues are important for *Tb*RIF4 function *in vivo*

Next we assessed whether *Tb*RIF4's conserved DnaQ exonuclease family DEDDh residues (Figure 1) were essential for *in vivo* function by mutating each amino acid to alanine in the context of the *Tb*RIF4-GFP complementation cassette, and introducing the resulting mutants into *rif4^−/−^* cells. With the exception of mutant E296A (Figure 4B, lane 5), the other proteins (D294A, D392A, H472A and D477A, lanes 4, 6, 7 and 8) were expressed close to the level of wild-type *Tb*RIF4-GFP (lane 3). Upon transfection of 5 µg α-tubulin dsRNA, which in wild-type cells results in approximately 84% degradation of target mRNA, in cells lacking *Tb*RIF4 or expressing mutants D294A, D392A or H472A there was very low or no detectable degradation of α-tubulin mRNA (Figure 2A). In contrast, transfection of dsRNA into cells expressing mutant D477A showed that at each dsRNA concentration tested, the cytoplasmic RNAi efficiency was reduced about 50% as compared to the wild-type level. Thus, it appeared that the D477 residue is not as important as the other predicted catalytic residues for *Tb*RIF4 activity *in vivo*. However, our results indicated that mutation of residues D294, D392 and H472 severely impaired the ability of cells to respond to dsRNA transfection. Similarly, nuclear RNAi as measured by RT-PCR of CIR147 repeats was partially restored in cells expressing mutant D477A (Figure 2C, lane 8), in agreement with the results of Figure 2A, whereas cells expressing mutants D294A, D392A and H472A appeared significantly impaired in nuclear RNAi (lanes 5–7). Finally, expression of active-site *Tb*RIF4 mutants D294A, D392A, H472A and D477A failed to restore *Tb*AGO1 to wild-type levels (Figure 4B, middle panel, lanes 4–8), although the amount of *Tb*AGO1 was increased as compared to *rif4^−/−^* cells.

### *Tb*RIF4 is part of a complex containing *Tb*AGO1 and siRNAs

**Figure 6:**
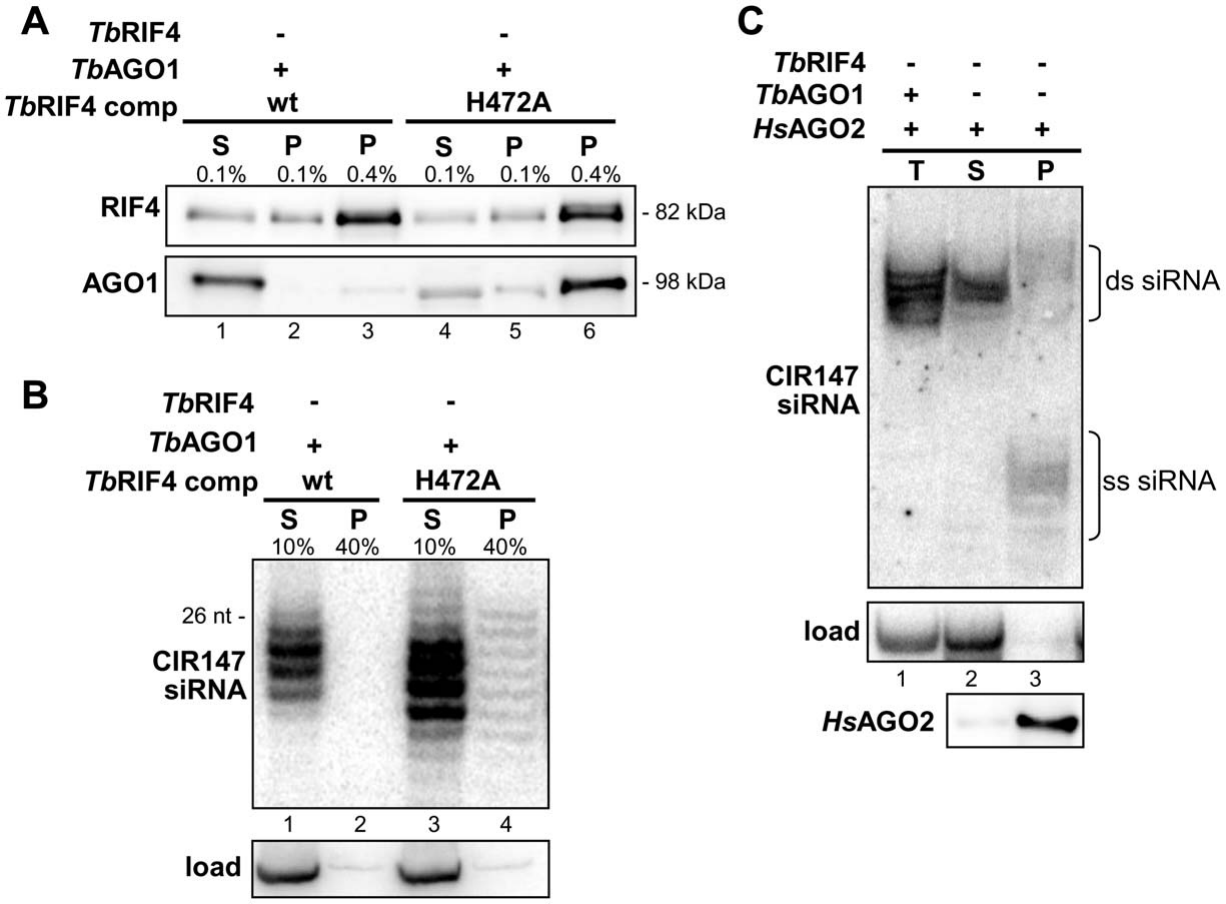
Interaction between *Tb*RIF4, *Tb*AGO1 and siRNAs. (*A*) Cytoplasmic extracts from *rif4^−/−^* cells expressing wt *Tb*RIF4-GFP (lanes 1–3) or mutant H472A *Tb*RIF4-GFP (lanes 4–6) were subjected to immunoprecipitation with anti-GFP antibody. 0.1% of the supernatant (S, lanes 1 and 4), 0.1% (lanes 2 and 5) and 0.4% (lanes 3 and 6) of the immunoprecipitated material were analyzed by Western blotting with a polyclonal anti-RIF4 antibody (top panel) or a polyclonal anti-*Tb*AGO1 antibody (bottom panel). The efficiency of immunoprecipitation is shown in the antibody titration experiment in Figure S7. (*B*) The presence of CIR147 siRNAs in the various samples as described in panel (*A*) was analyzed by Northern blot hybridization with a CIR147 probe. Load; hybridization to 5S rRNA. (*C*) Immunoprecipitated *Hs*AGO2 is associated with duplex and single-stranded siRNAs. Cytoplasmic extracts from cell lines expressing HA-FLAG-*Hs*AGO2 in an *ago1^−/−^:rif4^−/−^* genetic background, were immunoprecipitated with anti-FLAG antibodies. RNA associated with *Hs*AGO2 was separated on a native polyacrylamide gel and subjected to Northern hybridization with a CIR147 probe (upper panel). Total RNA from *rif4^−/−^* trypanosomes was used as a control for the position of duplex siRNAs (T, lane 1). Load; hybridization to 5S rRNA (middle panel). Equal amounts of the supernatant (S, lane 2) and immunoprecipitated material (P, lane 3) were analyzed by Western blotting with anti-HA antibody (*Hs*AGO2 panel).

The finding that siRNAs were not detectably associated with *Tb*AGO1 in *rif4^−/−^* cells could indicate a physical interaction between the two proteins. To test this hypothesis, we performed immunoprecipitations. Pull-downs of either wild-type or H472A *Tb*RIF4-GFP revealed the presence of *Tb*AGO1 in the immunoprecipitates (Figure 6A), with more *Tb*AGO1 in the H472A *Tb*RIF4-GFP sample compared to the wild-type sample (compare lanes 3 and 6, bottom panel). We next asked whether siRNAs could be immunoprecipitated with wild-type or H472A *Tb*RIF4-GFP (Figure 6B). In cells expressing wild-type *Tb*RIF4-GFP, CIR147 siRNAs were below the limit of detection (lane 2). In contrast, a fraction of CIR147 siRNAs were found in the immunoprecipitated material after pull-down of H472A *Tb*RIF4-GFP (lane 4). These results indicated that a proportion of a catalytically inactive *Tb*RIF4 was in a complex containing *Tb*AGO1 and siRNAs. We take these results as supportive evidence that *Tb*RIF4 and *Tb*AGO1 interact *in vivo*, either directly or indirectly, and that the interaction can be stabilized by mutation of the *Tb*RIF4 exonuclease active site, although the significance of the latter observation remains to be investigated further.

### *Tb*RIF4 substitutes for the slicer activity of human AGO2 in passenger strand removal

Our past and present data indicated that the transition from ds- to ss-siRNAs in *T. brucei* is uncoupled from *Tb*AGO1 slicer function and is carried out by the *Tb*RIF4 exonuclease. We previously reported that human AGO2 slicer (*Hs*AGO2), but not *Hs*AGO1, which lacks target transcript slicing activity, can partially complement *Tb*AGO1 null cells [17]. Since *Hs*AGO2 slicing activity is involved in the cleavage of both the passenger strand of duplex siRNAs and the target RNA [27], [33], we hypothesized that the restoration of the RNAi response by *Hs*AGO2 may occur by a *Tb*RIF4-independent mechanism. Thus, HA-FLAG-tagged *Hs*AGO2 was introduced into *rif4^−/−^*:*ago1^−/−^* cells. Expression of *Hs*AGO2 resulted in a partial cytoplasmic RNAi response measured by transfection with dsRNA (Figure 2A), as previously described for cells expressing *Hs*AGO2 and *Tb*RIF4, but nuclear RNAi was not restored (Figure 2C, lane 9), as evidenced by the accumulation of CIR147 transcripts. To monitor the physical state of the siRNAs, *Hs*AGO2 was immunoprecipitated and the associated siRNAs were analyzed by native gel electrophoresis (Figure 6C). In agreement with the partial RNAi response, a fraction of the siRNAs was single-stranded (lane 3). These results indicated that in trypanosomes expressing *Hs*AGO2 maturation of siRNAs from duplex- to ss-form, takes place independently of *Tb*RIF4 and underscored a model where *Tb*RIF4 in a complex with *Tb*AGO1 degrades the siRNA passenger strand, thus substituting for *Hs*AGO2 passenger cleavage activity in ss-siRNA biogenesis in *T. brucei*.

## Discussion

The availability of genome sequences from trypanosomatids that are either RNAi proficient or not, provided us with an opportunity to tease out genes specific to the RNAi mechanism. Quite surprisingly, the comparative genomics analysis revealed a limited set of five genes: the previously characterized *Tb*AGO1, *Tb*DCL1 and *Tb*DCL2, as well two additional candidates, *Tb*RIF4 and *Tb*RIF5. Here, we showed that both *Tb*RIF4 and *Tb*RIF5 are essential for RNAi, and carry out distinct functions. *Tb*RIF4 is implicated in the formation of a stable *Tb*AGO1/guide siRNA complex through conversion of duplex siRNAs to their single-stranded form, whereas *Tb*RIF5's function is less clearly defined due to a lack of recognizable domains, but this factor appears to be working in concert with *Tb*DCL1. The latter conclusion is based on the observations that *Tb*RIF5's ablation resulted in cytoplasmic RNAi deficiency and that the protein associated with *Tb*DCL1, the cytoplasmic Dicer. We therefore propose a role for *Tb*RIF5 as a cofactor in *Tb*DCL1-mediated processing of long dsRNA. It is unlikely that *Tb*RIF5 is involved in nuclear RNAi as CIR147 siRNAs, which are generated by *Tb*DCL2, are not affected by *Tb*RIF5 ablation, and we have recently shown that RNAi of small nucleolar RNAs similarly requires *Tb*DCL2, but is independent of *Tb*DCL1 [34]. The specific reduction of SLACS and ingi siRNAs in *Tb*RIF5 null cells points to the possibility that *Tb*RIF5 is required at some point in the biogenesis of such siRNAs in the cytoplasm, either by stimulating *Tb*DCL1 cleavage activity and/or by functioning as a bridge between *Tb*DCL1, the siRNAs and *Tb*AGO1. RNase III enzymes, such as Dicer and Drosha, in general require the assistance of dsRBP cofactors [12]. For instance, in Drosophila R2D2 forms a complex with DCR-2, enhances the incorporation of siRNAs into RISC [35] and acts as a sensor for strand asymmetry of siRNAs [36]. In human cells the interaction of DGCR8 with Drosha is essential for excision of pre-miRNAs from primary miRNA containing transcripts [37]. Although *Tb*RIF5 lacks a recognizable dsRNA binding domain, it may be that such a motif exists but is highly divergent.

*Tb*RIF4 contains a predicted 3′-5′ exonuclease domain. *In vivo Tb*RIF4 knockout results in both nuclear and cytoplasmic RNAi deficiency and in the accumulation of duplex siRNAs that are not detectably associated with *Tb*AGO1 by co-immunoprecipitations. As predicted from its domain structure, *Tb*RIF4-GST is endowed with *in vitro* 3′-5′ exonuclease activity, and processes duplex siRNAs. Complementation of *rif4^−/−^* cells with *Tb*RIF4-GFP carrying mutations of catalytic residues D294A, D392A and H472A, which also impair exonuclease activity *in vitro* (data not shown), did not restore RNAi competency of *Tb*RIF4 null cells, indicating that *Tb*RIF4 catalytic activity is required for RNAi function.

*Tb*RIF4 and *Tb*AGO1 interact, either directly or indirectly, and form a complex that can be recovered in greater amounts from mutant H472A *Tb*RIF4-GFP than from wild-type cells and contains a small proportion of siRNAs. Although at present it is unclear how this observation relates to *Tb*RIF4 function, it indicates that mutant *Tb*RIF4 becomes “stuck” in a complex with *Tb*AGO1. It is possible that the interaction between the two wild-type proteins is transient and that after the siRNA is converted to single-stranded form within this complex (either before or after loading into *Tb*AGO1), *Tb*RIF4 dissociates from *Tb*AGO1. *Tb*RIF4 on its own does not appear to show any strand selectivity; therefore, the strand removed is possibly determined by the orientation in which the duplex is presented by Dicer, as has been reported in Drosophila [38]. The use of chaperone proteins, for example heat shock proteins, in RISC loading has been reported in higher eukaryotes [39]–[42]. It is possible that *Tb*RIF4 acts in this way in addition to its role as an exonuclease.

We also observed that *Tb*AGO1 levels are diminished in cells lacking functional *Tb*RIF4. However, we note that there was no straightforward correlation between the amount of *Tb*AGO1 present in the various mutant cell lines and their degree of RNAi competency, suggesting that catalytically impaired *Tb*RIF4s can to some extent promote *Tb*AGO1 accumulation through a mechanism that is presently unknown. As *Tb*AGO1 mRNA levels are only marginally affected by *Tb*RIF4 deletion (data not shown), either *Tb*AGO1 translation is severely inhibited or the protein is made and rapidly degraded. So far inhibition of the proteasome and lysosome degradation pathways has not restored *Tb*AGO1 levels (unpublished observations), pointing to the possibility that translational control regulates the amount of *Tb*AGO1 that is synthesized.

*T. brucei* RIF4 shares with *Neurospora* QIP a C-terminal 3′-5′ exonuclease domain of the DnaQ superfamily. Although both proteins are essential RNAi factors and interact with the corresponding Argonaute, they are functionally distinct in several respects. First, in *Neurospora qip^ko^* cells duplex siRNAs are stably loaded into the AGO homolog QDE-2 and, after QDE-2 slicer-mediated cleavage of the passenger strand, remain bound to QDE-2 in duplex, nicked form [20]. In contrast, siRNAs can be successfully loaded into *Tb*AGO1 and *Tb*RIF4 can accomplish the transition from double- to single-stranded siRNAs in the absence of *Tb*AGO1 slicer activity [21], [22]. Secondly, we showed that *Tb*RIF4 activity is required for *Tb*AGO1 protein accumulation. This finding is not mirrored in *Neurospora* or *Drosophila*, where mutation of passenger strand fragment removal factors QIP or C3PO has no effect on the corresponding AGO levels [20], [28]. In light of these data, it is evident that *Tb*RIF4 and *Nc*QIP are functionally distinct RNAi factors.

In trypanosomes expressing *Hs*AGO2, maturation of siRNAs takes place independently of *Tb*RIF4, suggesting a model where *Tb*RIF4 substitutes for AGO slicing activity in the generation of ss-siRNAs. This would represent a departure from strategies of RISC maturation in higher eukaryotes, which favor slicer activity marking the passenger strand for degradation, although bypass pathways can operate in the absence of slicer activity [27], [43], [44].

Taken together with our earlier studies, the results presented here provide a detailed depiction of the core RNAi machinery in the early divergent parasitic protozoan *T. brucei*. In these organisms, the RNAi pathway is initiated by two distinct Dicer-like enzymes, namely *Tb*DCL1, mostly found in the cytoplasm [15], and *Tb*DCL2, a mainly nuclear Dicer responsible for processing dsRNAs originating from retroposons and satellite-like repeats [16]. siRNAs generated by both Dicers are channelled into a single member of the Argonaute family of proteins, namely *Tb*AGO1 [13], [14]. The identification and characterization of *Tb*RIF4 and *Tb*RIF5 most likely completes the collection of core components shared by RNAi-positive trypanosomatids. However, it is likely that, as described in other organisms, other cellular factors interact with the core RNAi machinery. Some of these factors may perform housekeeping functions, for instance the Hsp90/70 family of chaperons that functions in RISC assembly in higher eukaryotes, and be present in all trypanosomatids irrespective of their RNAi proficiency, while others may be organism-specific. Indeed, we have recently discovered that the *T. brucei* homologue of the plant HEN1 2′-*O* methyltransferase [45] is required for siRNA 3′ end modification (unpublished observation). However, siRNA modification is not an absolute requirement for trypanosomatid RNAi, as in *L. braziliensis* the HEN1 gene is absent, and deletion of *Tb*HEN1 is still compatible with a robust RNAi response (unpublished observations). It could be argued at this point that attempting to reconstitute RNAi in RNAi-deficient pathogens is quite daunting with a minimum of five core components. However, our past and current mechanistic studies have highlighted that not all components are necessary. In particular, we have shown previously that *Tb*DCL2-deficient cells are more responsive to RNAi triggers than wild-type cells [16] suggesting that this factor does not need to be included in a reconstitution attempt. In addition, in the present study we have shown that human AGO2 can functionally replace both *Tb*AGO1 and *Tb*RIF4, indicating that the *Tb*RIF4 function can be bypassed by using an appropriate AGO protein. It will be interesting to see whether human AGO2 can similarly function in *L. braziliensis*. The factor remaining a mystery is *Tb*RIF5 and its function will be challenging to ascertain due to a complete lack of a possible functional indicator. Nevertheless, the available proof of principle that RNAi can be reconstituted in *S. cerevisiae* by using only AGO1 and DCR1 from *S. castelli* [9] in combination with our functional studies raises the intriguing possibility that a similar strategy might be applicable in RNAi-negative trypanosomatids.

## Materials and Methods

### Standard methods

Previously published procedures were followed for culturing trypanosome YTat 1.1 cells [46], generation of knockout cell lines by PCR-based methods [47], Northern blots of total RNA [32], Western blots [14], S100 preparation [32], immunoprecipitations [14], and the Dicer *in vitro* assay [16].

### RT-PCR

5 µg of total RNA from various cell lines was treated with 2 units of DNase RQ (Promega), phenol extracted and ethanol precipitated. DNase-treated RNA was used in a reverse transcription reaction using the manufacturer's protocol for the Superscript II enzyme (Invitrogen), phenol extracted, ethanol precipitated, and resuspended in 100 µl water. Mock reactions without the reverse transcriptase enzyme were carried out in parallel. 22 cycles of PCR were performed using the Phusion enzyme (Finnzyme) and the GC buffer provided, according to manufacturer's instructions: 50°C annealing temperature, 10 sec extension time for the CIR147 reactions, 30 sec extension time for the histone 4 reactions.

### Preparation of small RNAs

Small RNAs were extracted from S100 extracts by incubation in polysome buffer plus 20 mM EDTA, 2% SDS and 0.1 µg/µl proteinase K for 1 hour at room temperature. An equal volume of phenol and 1/10 volume 20× SET [pH 7.4] were added and the mixture was centrifuged at 13,200× g for 5 min. The supernatant was incubated with 600 µl isopropanol and 30 µg GlycoBlue (Ambion) at −20°C for 30 min and centrifuged at 13,200× g for 10 min at 4°C. The pellet was washed with 70% ethanol, dried and resuspended in water (for native gels) or urea loading buffer (for denaturing gels). RNA was transferred to a positively-charged nylon membrane (Hybond N+, GE Healthcare) and UV cross-linked at 120 mJ/cm^2^. The membrane was incubated in boiling water, which was allowed to cool to room temperature, twice prior to probe hybridization. This treatment significantly improved detection of dsRNA.

### Preparation of probes

DNA oligos were 5′ end-labelled with T4 polynucleotide kinase (NEB) using [γ-^32^P]ATP and purified using P6 columns (BioRad). Hybridizations were carried out in ExpressHyb solution (Clontech) overnight at 30°C. Washes in 2× SSC, 0.2% SDS were carried out at room temperature. Blots were analyzed by PhosphorImager.

### Purification of recombinant protein

*Tb*RIF4 was cloned into the pGEX4T-1 vector. Inductions of 500 ml DH5α bacteria were carried out by adding 0.2 mM isopropyl β-D-1-thiogalactopyranoside (IPTG) overnight at 16°C. Cells were washed in PBS containing protease inhibitors, lysed by sonication in 5 ml PBS containing protease inhibitors and GST-*Tb*RIF4 was purified using a SpinTrap column (GE). Proteins were eluted in 50 mM Tris-HCl [pH 8.0], 10 mM glutathione, before being applied to a desalting column and stored in assay buffer (20 mM HEPES-KOH [pH 7.9], 150 mM sucrose, 20 mM potassium L-glutamate, 3 mM MgCl_2_, 2 mM DTT).

### Exonuclease assays

Exonuclease assays were carried out for 30 min at 28°C in 10 µl assay buffer including RNase Inhibitor (Roche) and 0.01 µg/µl bovine tRNA. Reactions were terminated by the addition of 4× SET, 20 mM EDTA, 30 µg GlycoBlue and isopropanol, stored at −20°C for 30 min and spun at 13,200× g for 10 min at 4°C. The pellet was washed with 70% ethanol, dried and resuspended in urea loading buffer. Samples were resolved on a 20% denaturing polyacrylamide gel and analyzed by PhosphorImager.

## Supporting Information

Figure S1 ***Tb*** **RIF4 sequence analysis.** (*A*) Sequence alignment of *Tb*RIF4 protein sequences from *T. brucei* (Tbru), *T. congolense* (Tcon), *T. vivax* (Tviv) and *L. (V.) braziliensis* (Lbra) created using ClustalX (www.clustal.org) and shaded using boxshade (http://www.ch.embnet.org/software/BOX_form.html). Conserved motifs are outlined, and conserved active site residues are indicated by asterisks. (*B*) Ribbon diagram of the secondary structure of *E. coli* DnaQ (left) adapted from [25] and of the predicted model of the exonuclease domain of *Tb*RIF4 (right) using the alignment interface of SWISS-MODEL [24].
(TIF)

Figure S2 ***Tb*** **RIF5 sequence analysis.** Sequence alignment of *Tb*RIF5 protein sequences from *T. brucei* (Tbru), *T. congolense* (Tcon), *T. vivax* (Tviv) and *L. (V.) braziliensis* (Lbra) created using ClustalX (www.clustal.org) and shaded using boxshade (http://www.ch.embnet.org/software/BOX_form.html).
(TIF)

Figure S3 ***Tb*** **RIF4 and** ***Tb*** **RIF5 knockout and complementation cell lines.** (*A*) *rif4^−/−^* cells do not express *Tb*RIF4 mRNA (top panel) or protein (bottom panel). Total RNA isolated from various cell lines, as indicated above each lane, was resolved by formaldehyde-agarose gel electrophoresis and analyzed by Northern blot hybridization with a *Tb*RIF4-specific probe (top panel). Load; α-tubulin hybridization. Total protein extracts were resolved by SDS-PAGE and probed with a polyclonal anti-*Tb*RIF4 antibody (third panel). Load; a cross-reacting band. *(B) rif5^−/−^* cells do not express *Tb*RIF5 mRNA (top panel). Total RNA isolated from various cell lines, as indicated above each lane, was resolved by formaldehyde-agarose gel electrophoresis and analyzed by Northern blot hybridization with a RIF5-specific probe (top panel). Load; α-tubulin hybridization. Ten-fold less RNA was loaded in the *rif5c* lane compared to the other two lanes.
(TIF)

Figure S4 **Physical association of** ***Tb*** **DCL1 and** ***Tb*** **RIF5.** Cytoplasmic extracts from *rif4^−/−^* cells expressing DCL1-FLAG-BB2 and RIF5-HA-BB2 were subjected to immunoprecipitation with anti-FLAG antibody (upper panel) or anti-HA antibody (lower panel). Equal numbers of cell equivalents of the input (I), supernatant (S), and immunoprecipitated material (P) were analyzed by Western blotting with a monoclonal anti-BB2 antibody.
(TIF)

Figure S5 **siRNAs in** ***rif4^−/−^*** **trypanosomes are double-stranded.** (*A*) Native gel analysis of siRNAs in *rif4^−/−^* cells. RNA isolated from a *rif4^−/−^* S100 extract was resolved on a native polyacrylamide gel after heating the samples at a range of temperatures as indicated above each lane for 2 minutes prior to electrophoresis and analyzed by Northern blotting with an ingi probe. (*B*) siRNAs in *rif4^−/−^* cells are protected from digestion with RNase. S100-derived RNAs from wild-type (lanes 1–3) and *rif4^−/−^* (lanes 4–6) cells were treated with RNase T1 (5 U/ml) and RNase A (20 µg/ml) for 1 hr at 25°C, either without (lanes 2 and 5) or with (lanes 3 and 6) denaturation by boiling prior to enzyme addition. RNAs incubated with buffer alone (lanes 1 and 4) were included as a control. Following digestion with proteinase K, the samples were electrophoresed on a 16% denaturing gel and analyzed by Northern hybridization with a CIR147 probe (top panel). Load; hybridization to 5S rRNA (bottom panel).
(TIF)

Figure S6 **Recombinant** ***Tb*** **RIF4 3′-5′ exonuclease activity** ***in vitro*** **.** (*A*) Purification of GST-*Tb*RIF4. 30 pmol of recombinant GST-*Tb*RIF4 was resolved by SDS-PAGE and stained with Coomassie Brilliant Blue. (*B*) Time-course of GST-*Tb*RIF4 activity. 1 pmol of a 25 nt-long, 5′-end labelled synthetic dsRNA was incubated with 30 pmol GST-RIF4 for 1 to 30 min (lanes 2–7). The activity of GST-*Tb*RIF4 carrying mutation H472A incubated for 30 min is shown in lane 8, and a no-protein control is in lane 1. (*C*) GST-*Tb*RIF4 activity is not restricted to one of the duplex RNA strands. 1 pmol of synthetic siRNA-315 labelled at the 5′ end of either the “sense” or “antisense” strand was incubated with wild-type GST-*Tb*RIF4 (wt; lanes 2 and 5), GST-*Tb*RIF4 carrying mutation H472A (H472A; lanes 3 and 6) or buffer alone (lanes 1 and 4). (*D*) Action of GST-*Tb*RIF4 on siRNA-like substrates with shorter 3′ overhangs. 1 pmol of a 25 nt-long, 5′-end labelled synthetic dsRNA with 3′ overhangs of 2, 1 or 0 nt was incubated with 0.04 to 25 pmol GST-*Tb*RIF4 (lanes 2–6, 8–12 and 14–18) or buffer alone (lanes 1, 7 and 13). (*E*) GST-*Tb*RIF4 preferentially cleaves double-stranded RNA. 1 pmol synthetic ssRNA (lanes 1 and 2) or dsRNA (lanes 3 and 4) was incubated with (+; lanes 2 and 4) or without (−; lanes 1 and 3) 30 pmol wild-type GST-*Tb*RIF4. (*F*) GST-*Tb*RIF4 activity is abolished by addition of EDTA. 1 pmol synthetic dsRNA was incubated with 30 pmol wild-type GST-RIF4 (wt; lanes 2 and 5), GST-*Tb*RIF4 carrying mutation H472A (H472A; lanes 3 and 6) or buffer alone (lanes 1 and 4). 5 mM EDTA was added in the reactions for lanes 4–6. (*G*) GST-*Tb*RIF4 activity requires a free 3′ end. 1 pmol synthetic dsRNA either without (lanes 1–3) or with (lanes 4–6) a terminal 2′,3′-dideoxycytidine residue was incubated with 30 pmol GST-*Tb*RIF4 (wt; lanes 2 and 5), GST-*Tb*RIF4 carrying mutation H472A (H472A; lanes 3 and 6) or buffer alone (lanes 1 and 4).
(TIF)

Figure S7 **Efficient pull-down of** ***Tb*** **RIF4-GFP.** Cytoplasmic extracts from *rif4^−/−^* cells expressing wt *Tb*RIF4-GFP were subjected to immunoprecipitation with anti-GFP antibody. Equal volumes of the input (I), supernatant (S) and immunoprecipitated material (P) were analyzed by Western blotting with a polyclonal anti-*Tb*RIF4 antibody.
(TIF)
