# Supplementary figures and images for: Comparative Genomics Reveals Two Novel RNAi Factors in Trypanosoma brucei and Provides Insight into the Core Machinery
Source: PLoS Pathog. 2012 May 24;8(5):e1002678. doi: 10.1371/journal.ppat.1002678 (PMC3359990; doi:10.1371/journal.ppat.1002678)

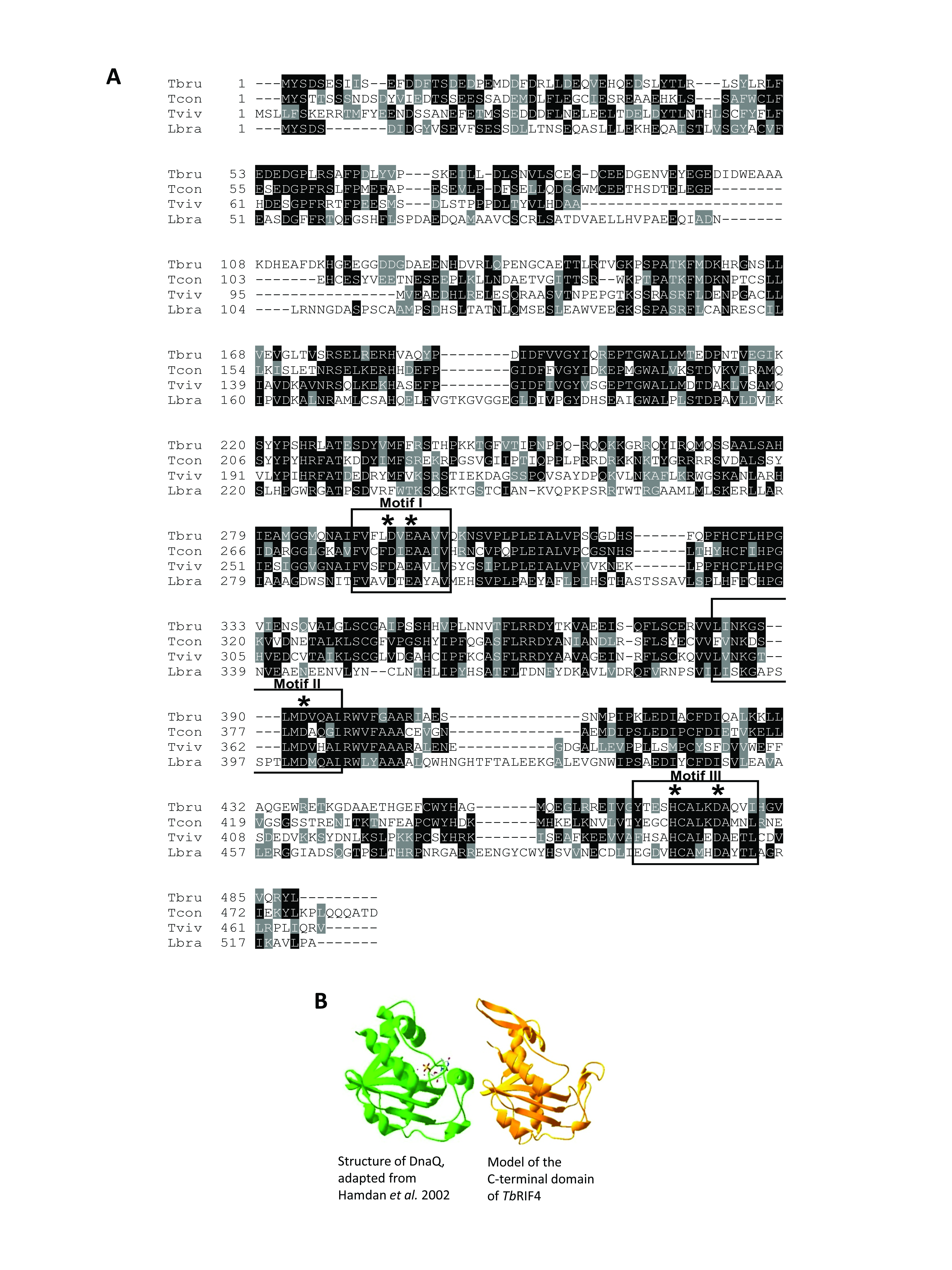

Supplement: Figure S1 — Tb RIF4 sequence analysis. (A) Sequence alignment of TbRIF4 protein sequences from T. brucei (Tbru), T. congolense (Tcon), T. vivax (Tviv) and L. (V.) braziliensis (Lbra) created using ClustalX (www.clustal.org) and shaded using boxshade (http://www.ch.embnet.org/software/BOX_form.html). Conserved motifs are outlined, and conserved active site residues are indicated by asterisks. (B) Ribbon diagram of the secondary structure of E. coli DnaQ (left) adapted from [25] and of the predicted model of the exonuclease domain of TbRIF4 (right) using the alignment interface of SWISS-MODEL [24]. (TIF) [file ppat.1002678.s001.tif]

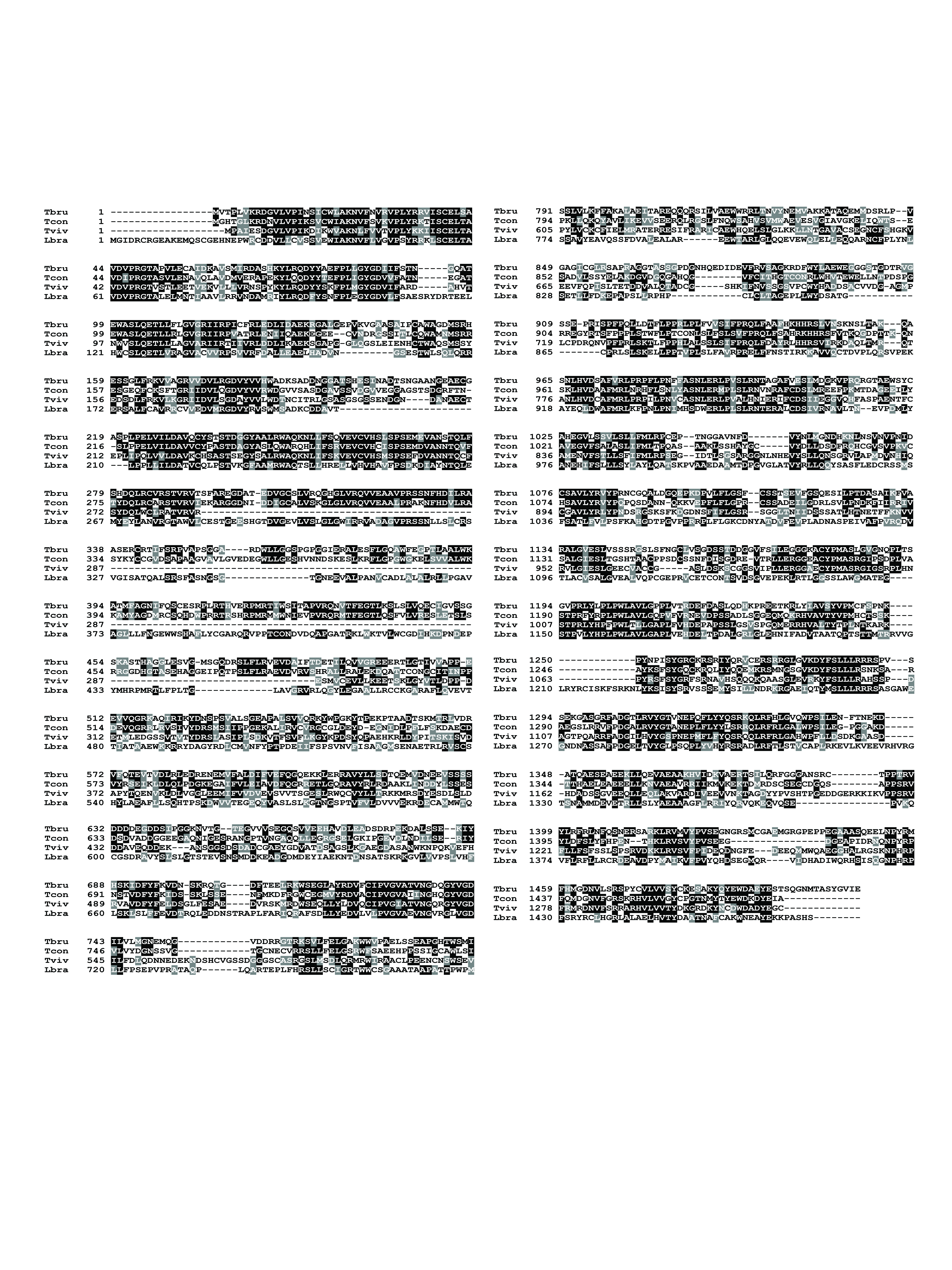

Supplement: Figure S2 — Tb RIF5 sequence analysis. Sequence alignment of TbRIF5 protein sequences from T. brucei (Tbru), T. congolense (Tcon), T. vivax (Tviv) and L. (V.) braziliensis (Lbra) created using ClustalX (www.clustal.org) and shaded using boxshade (http://www.ch.embnet.org/software/BOX_form.html). (TIF) [file ppat.1002678.s002.tif]

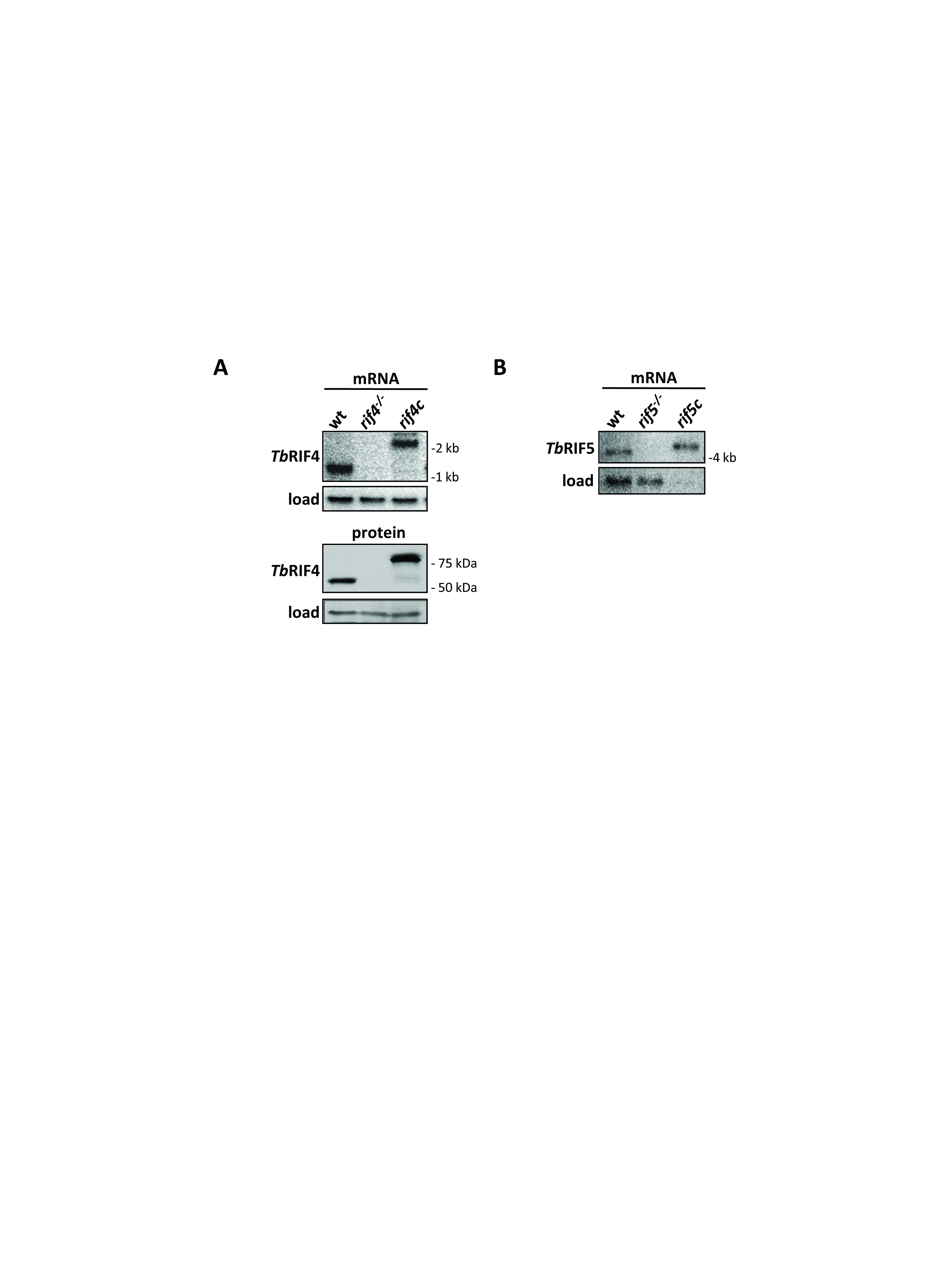

Supplement: Figure S3 — Tb RIF4 and Tb RIF5 knockout and complementation cell lines. (A) rif4−/− cells do not express TbRIF4 mRNA (top panel) or protein (bottom panel). Total RNA isolated from various cell lines, as indicated above each lane, was resolved by formaldehyde-agarose gel electrophoresis and analyzed by Northern blot hybridization with a TbRIF4-specific probe (top panel). Load; α-tubulin hybridization. Total protein extracts were resolved by SDS-PAGE and probed with a polyclonal anti-TbRIF4 antibody (third panel). Load; a cross-reacting band. (B) rif5−/− cells do not express TbRIF5 mRNA (top panel). Total RNA isolated from various cell lines, as indicated above each lane, was resolved by formaldehyde-agarose gel electrophoresis and analyzed by Northern blot hybridization with a RIF5-specific probe (top panel). Load; α-tubulin hybridization. Ten-fold less RNA was loaded in the rif5c lane compared to the other two lanes. (TIF) [file ppat.1002678.s003.tif]

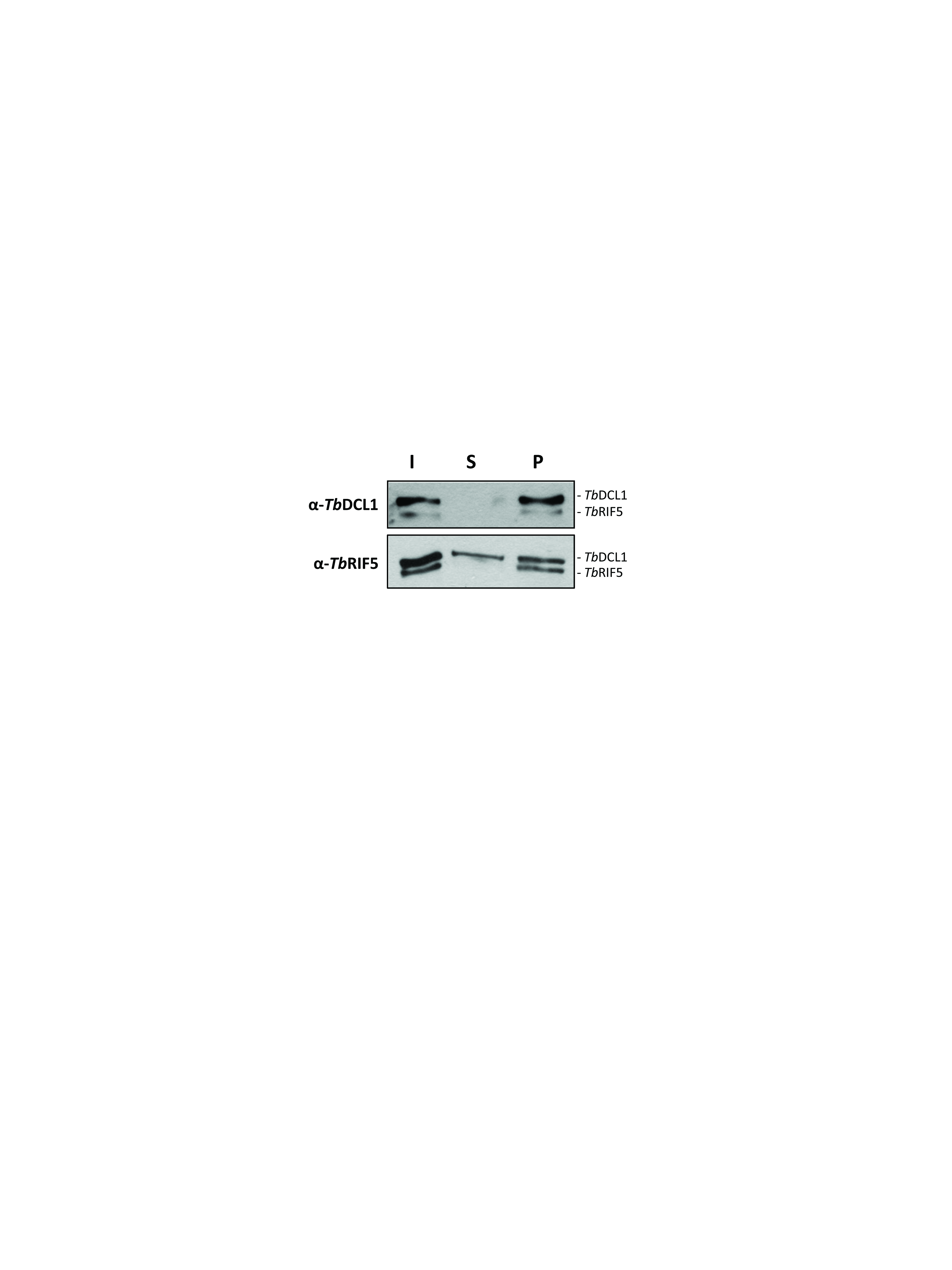

Supplement: Figure S4 — Physical association of Tb DCL1 and Tb RIF5. Cytoplasmic extracts from rif4−/− cells expressing DCL1-FLAG-BB2 and RIF5-HA-BB2 were subjected to immunoprecipitation with anti-FLAG antibody (upper panel) or anti-HA antibody (lower panel). Equal numbers of cell equivalents of the input (I), supernatant (S), and immunoprecipitated material (P) were analyzed by Western blotting with a monoclonal anti-BB2 antibody. (TIF) [file ppat.1002678.s004.tif]

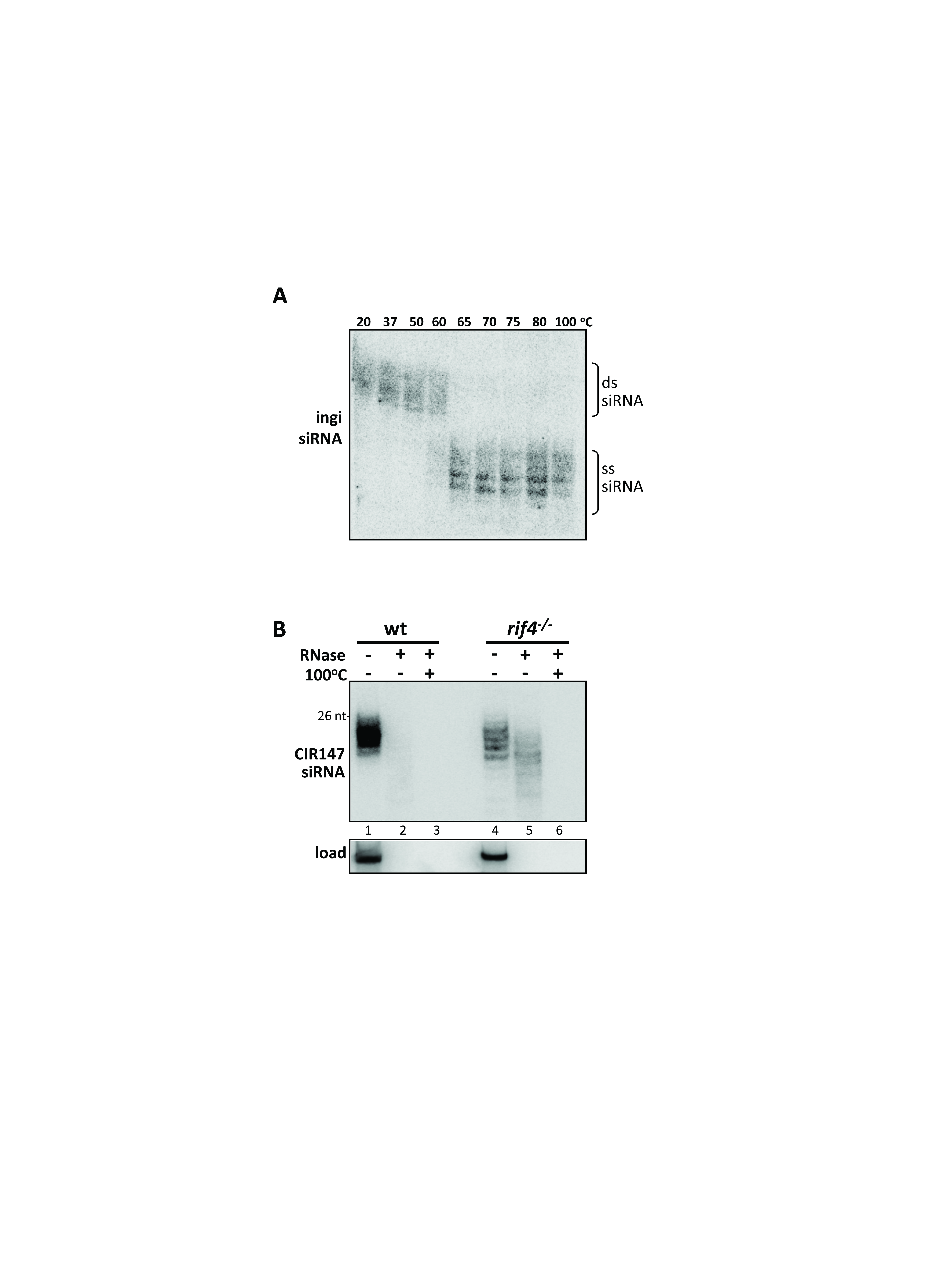

Supplement: Figure S5 — siRNAs in rif4−/− trypanosomes are double-stranded. (A) Native gel analysis of siRNAs in rif4−/− cells. RNA isolated from a rif4−/− S100 extract was resolved on a native polyacrylamide gel after heating the samples at a range of temperatures as indicated above each lane for 2 minutes prior to electrophoresis and analyzed by Northern blotting with an ingi probe. (B) siRNAs in rif4−/− cells are protected from digestion with RNase. S100-derived RNAs from wild-type (lanes 1–3) and rif4−/− (lanes 4–6) cells were treated with RNase T1 (5 U/ml) and RNase A (20 µg/ml) for 1 hr at 25°C, either without (lanes 2 and 5) or with (lanes 3 and 6) denaturation by boiling prior to enzyme addition. RNAs incubated with buffer alone (lanes 1 and 4) were included as a control. Following digestion with proteinase K, the samples were electrophoresed on a 16% denaturing gel and analyzed by Northern hybridization with a CIR147 probe (top panel). Load; hybridization to 5S rRNA (bottom panel). (TIF) [file ppat.1002678.s005.tif]

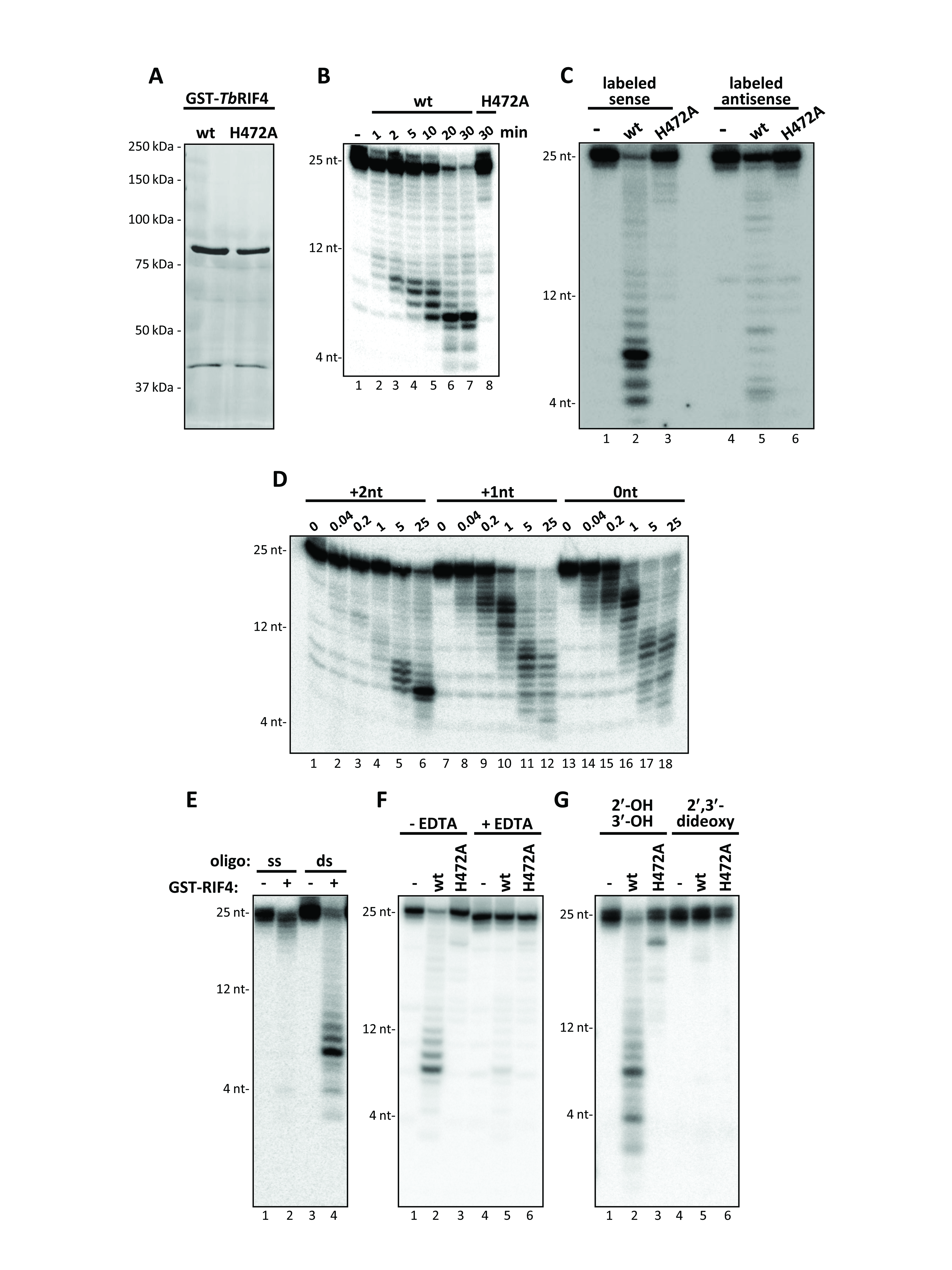

Supplement: Figure S6 — Recombinant Tb RIF4 3′-5′ exonuclease activity in vitro . (A) Purification of GST-TbRIF4. 30 pmol of recombinant GST-TbRIF4 was resolved by SDS-PAGE and stained with Coomassie Brilliant Blue. (B) Time-course of GST-TbRIF4 activity. 1 pmol of a 25 nt-long, 5′-end labelled synthetic dsRNA was incubated with 30 pmol GST-RIF4 for 1 to 30 min (lanes 2–7). The activity of GST-TbRIF4 carrying mutation H472A incubated for 30 min is shown in lane 8, and a no-protein control is in lane 1. (C) GST-TbRIF4 activity is not restricted to one of the duplex RNA strands. 1 pmol of synthetic siRNA-315 labelled at the 5′ end of either the “sense” or “antisense” strand was incubated with wild-type GST-TbRIF4 (wt; lanes 2 and 5), GST-TbRIF4 carrying mutation H472A (H472A; lanes 3 and 6) or buffer alone (lanes 1 and 4). (D) Action of GST-TbRIF4 on siRNA-like substrates with shorter 3′ overhangs. 1 pmol of a 25 nt-long, 5′-end labelled synthetic dsRNA with 3′ overhangs of 2, 1 or 0 nt was incubated with 0.04 to 25 pmol GST-TbRIF4 (lanes 2–6, 8–12 and 14–18) or buffer alone (lanes 1, 7 and 13). (E) GST-TbRIF4 preferentially cleaves double-stranded RNA. 1 pmol synthetic ssRNA (lanes 1 and 2) or dsRNA (lanes 3 and 4) was incubated with (+; lanes 2 and 4) or without (−; lanes 1 and 3) 30 pmol wild-type GST-TbRIF4. (F) GST-TbRIF4 activity is abolished by addition of EDTA. 1 pmol synthetic dsRNA was incubated with 30 pmol wild-type GST-RIF4 (wt; lanes 2 and 5), GST-TbRIF4 carrying mutation H472A (H472A; lanes 3 and 6) or buffer alone (lanes 1 and 4). 5 mM EDTA was added in the reactions for lanes 4–6. (G) GST-TbRIF4 activity requires a free 3′ end. 1 pmol synthetic dsRNA either without (lanes 1–3) or with (lanes 4–6) a terminal 2′,3′-dideoxycytidine residue was incubated with 30 pmol GST-TbRIF4 (wt; lanes 2 and 5), GST-TbRIF4 carrying mutation H472A (H472A; lanes 3 and 6) or buffer alone (lanes 1 and 4). (TIF) [file ppat.1002678.s006.tif]

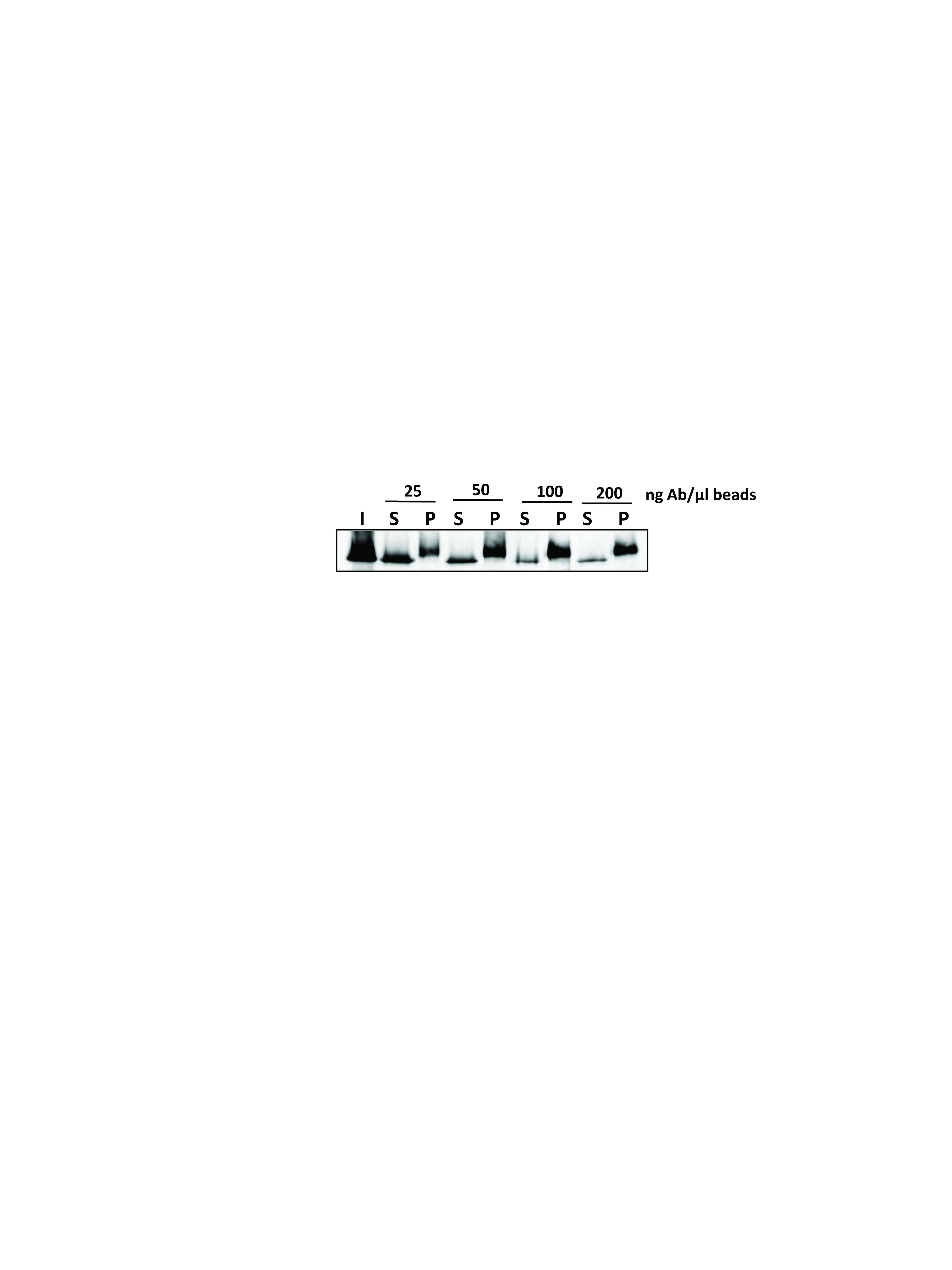

Supplement: Figure S7 — Efficient pull-down of Tb RIF4-GFP. Cytoplasmic extracts from rif4−/− cells expressing wt TbRIF4-GFP were subjected to immunoprecipitation with anti-GFP antibody. Equal volumes of the input (I), supernatant (S) and immunoprecipitated material (P) were analyzed by Western blotting with a polyclonal anti-TbRIF4 antibody. (TIF) [file ppat.1002678.s007.tif]
